# Difficulties at Lambeth Infirmary

**Published:** 1920-08-14

**Authors:** 


### Difficulties at Lambeth Infirmary.

Lambeth Board of Guardians have again attempted to tackle the unfortunate conditions prevailing at the infirmary. These are chiefly due to the absence of adequate residential accommodation for the officers, so that it has not been possible to reduce the excessive number of hours they are required to be on duty. This condition was aggravated because all the non-resident officers in the infirmary have been brought within the scope of the 48-hour week. The medical officer and the matron have presented reports to the Infirmary Committees on these matters, in the endeavour, while not immediately reducing the working hours, to give the nurses greater comfort and more opportunities of recreation on the premises. The Board has adopted the principle of the 48-hour week to its nurses, and to carry this out has again approached the Ministry of Health to give a final decision on the proposal to employ non-resident probationer nurses. The Guardians learnt that the payment of overtime to officers beyond the 48-hour week was a system which had been adopted by some public bodies, and the Committee, which had considered this matter and was unable to recommend payment for overtime, was asked to reconsider its view.

An extraordinary meeting of the Board was called to discuss the question of purchasing some houses which could be adapted for accommodation for the nurses, and there is reason to believe that the Guardians will be able to acquire them and make them comfortable for the staff. Meantime, the Guardians have decided to provide additional recreation-rooms over the Board Room and the clerk's general offices, close to the present nurses' quarters. Other alterations are to be carried out at the mess-room, kitchen, and adjoining offices which will, to a great extent, remove many of the existing inconveniences. It is strange, however, that many years have passed before these unfortunate conditions were discovered. An assurance was given that there are now no nurses at the infirmary on duty twelve hours a day, and before long probably Lambeth Board of Guardians will be able to see that all their officers work only the 48-hour week.

